# Detrimental Effects of a Retrieval-Extinction Procedure on Nicotine Seeking, but Not Cocaine Seeking

**DOI:** 10.3389/fnbeh.2019.00243

**Published:** 2019-10-15

**Authors:** Roeland F. Struik, Taco J. De Vries, Jamie Peters

**Affiliations:** ^1^Department of Anatomy & Neurosciences, Amsterdam Neuroscience, VU University Medical Center, Amsterdam, Netherlands; ^2^Department of Anesthesiology, University of Colorado School of Medicine, Anschutz Medical Campus, Aurora, CO, United States

**Keywords:** retrieval-extinction, reconsolidation, spontaneous recovery, nicotine, cocaine, self-administration

## Abstract

Retrieval-extinction memory reactivation procedures have been used to prevent the return of learned fear and drug seeking in preclinical models. These procedures first reactivate the original memory with a brief cue exposure (i.e., retrieval) session, and then disrupt memory reconsolidation by conducting extinction training within the reconsolidation window. The original memory is thought to be updated with the new information conveyed by extinction learning, resulting in a persistent therapeutic effect beyond that observed with extinction training alone (i.e., no retrieval). Here, we attempted to replicate the therapeutic effects on cocaine seeking reported by Xue et al. (2012), and extend these findings to nicotine seeking. Rats self-administered either cocaine or nicotine with contingent cues for weeks, and were then divided into two groups. The retrieval group underwent a 10-min retrieval session wherein drug cues were available, but drug was not. Ten minutes later, they were allowed to continue cue extinction training for an additional 60 min. The no retrieval group underwent a contiguous 70-min cue extinction session. These procedures continued for weeks, followed by a test for spontaneous recovery of drug seeking. No group differences were observed on any measure of cocaine seeking, although both groups exhibited extinction and spontaneous recovery. By contrast, for nicotine seeking, the retrieval group exhibited resistance to extinction, an effect that persisted on the spontaneous recovery test. These findings underscore the importance of drug type in the outcome of retrieval-extinction procedures and moreover indicate that retrieval-extinction procedures can be detrimental to nicotine seeking.

## Introduction

Cue exposure therapy has been used clinically in the treatment of both pathological fear and substance use disorders (Kaplan et al., 2010). During these behavioral therapy sessions, patients are typically repeatedly presented with visual, auditory, and/or tactile cues associated with the fear or drug memory, and over time learn to dissociate the cues from the feelings/cravings they induce. This learning process is referred to as extinction, and the pathological emotional and/or behavioral response is extinguished through the formation of a novel therapeutic memory trace (Quirk and Mueller, 2007). Unfortunately, extinction therapy has not been very effective on its own, especially for addiction treatment (Niaura et al., 1999; Kantak and Nic Dhonnchadha, 2011; Hone-Blanchet et al., 2014). An alternative approach is to attempt to weaken or erase the original memory associated with conditioned drug cues through a process of reconsolidation blockade (Taylor et al., 2008; Torregrossa and Taylor, 2012).

Reconsolidation is the process whereby memories enter into a labile state upon retrieval, typically by presentation of reminder cues (Schwabe et al., 2014). Once the memory enters this labile state, it is subject to disruption by pharmacological agents such as protein synthesis inhibitors (Nader et al., 2000; Tronson and Taylor, 2007). If the memory is disrupted during this sensitive period, it can be permanently weakened, even erased, along with the pathological behavioral response that is the goal of treatment (Soeter and Kindt, 2011). Unfortunately, protein synthesis inhibitors like anisomycin and related pharmacological tools for reconsolidation blockade cannot be administered systemically in humans due to toxicity, although some success has been demonstrated with the beta adrenergic receptor antagonist propranolol in both fear as well as cocaine and nicotine addiction disorders (Soeter and Kindt, 2010; Otis and Mueller, 2011; Saladin et al., 2013; Xue et al., 2017). Circumventing the need for such pharmacological approaches, Monfils et al. (2009) made a revolutionary discovery that memory reconsolidation could be disrupted by purely behavioral means.

Monfils et al. (2009) demonstrated that simply conducting extinction training, an extended cue exposure session, after brief fear memory retrieval was sufficient to disrupt the fear memory and prevent the return of fear. This procedure is thought to update the original fear memory with the “safety” memory learned during extinction, while the original memory is undergoing reconsolidation (Monfils et al., 2009; Quirk et al., 2010). Since this original report, many others have observed similar therapeutic effects of such retrieval-extinction memory reactivation procedures on fear, including translational work in humans (Quirk et al., 2010; Schiller et al., 2010; Flavell et al., 2011). However, others have reported null effects and/or challenged the notion that reconsolidation is necessary for the observed behavioral outcomes (Chan et al., 2010; Costanzi et al., 2011; Kindt and Soeter, 2013). Corroborating and conflicting reports have been reported for aversive and appetitive behaviors, in both preclinical and clinical settings (Flavell et al., 2011; Ma et al., 2012; Auber et al., 2013; Kredlow et al., 2016; Elsey et al., 2018), making the retrieval-extinction procedure one of the most controversial therapies with high treatment potential for multiple neuropsychiatric disorders.

In a seminal article by Xue et al. (2012), the retrieval-extinction memory reactivation procedure was adapted and applied to preclinical models of drug self-administration. Addiction, unlike fear, develops gradually over a prolonged period of drug exposure. Similarly, extinction learning typically requires repetitive training to extinguish the drug-seeking response. Thus, Xue et al. (2012) conducted daily retrieval-extinction sessions over a period of weeks, each with a brief retrieval (i.e., extinction) session separated from a longer extinction session by a 10 min interval. In cocaine-seeking animals, these procedures successfully facilitated the rate of extinction and reduced cocaine-primed reinstatement, renewal, and spontaneous recovery after a 28-day abstinence period. They also demonstrated that the therapeutic effect on drug-primed reinstatement extended to heroin-seeking animals, and were able to further extend their findings to human heroin users, using a similar 10-min interval between retrieval and extinction of visual heroin cues in a clinical setting (Xue et al., 2012).

Given the striking efficacy of these retrieval-extinction procedures across drug classes and from preclinical to clinical paradigms, we designed experiments with two goals: (1) to replicate these findings in cocaine-seeking animals; and (2) to extend these findings to nicotine-seeking animals. Nicotine conditioned cues are unique in that they are necessary and sufficient to sustain nicotine-seeking, even in the absence of nicotine (Caggiula et al., 2001; Cohen et al., 2005; Liu et al., 2008). Nonetheless, cue-maintained responding of nicotine-seeking can be extinguished and reduces withdrawal-related incubation of craving in preclinical abstinence models (Markou et al., 2018). Thus, retrieval-extinction procedures could be especially beneficial for reducing relapse associated with exposure to nicotine cues. Preliminary success has been reported using retrieval-extinction procedures to reduce nicotine seeking in a preclinical model in rats (Auber et al., 2014), as well as a recent clinical study in human smokers (Germeroth et al., 2017).

## Materials and Methods

### Subjects

Male Wistar rats (*n* = 48) obtained from Harlan CPB (Horst, The Netherlands), weighing between 280 and 300 grams on arrival, were housed in a temperature (21 ± 1°C) and humidity-controlled room (55 ± 15%) on a reverse 12 h diurnal schedule (lights off: 07:00; lights on: 19:00). Food and water was available *ad libitum* in the home cage. All experimental procedures were performed during the dark phase of the cycle. Animals were initially kept in pairs but were housed individually following surgery in Makrolon type III cages. Experiments and procedures were approved by the Animal Experiments Committee of the VU University, Amsterdam, The Netherlands.

### Surgery

Intravenous catheter surgeries were performed to allow drug self-administration. Catheters were assembled from a cannula connector pedestal (Plastics One Inc., Minneapolis, MN, USA) connected to a 95 mm silicone catheter (0.3 mm inner diameter × 0.6 mm outer diameter) and a 6 mm protective sleeve of polyethylene tubing (0.75 mm inner diameter × 1.45 mm outer diameter). Following arrival, rats were habituated to the animal facility for 1 week. The surgical procedure was executed as reported previously (De Vries et al., 1999) under isoflurane gas anesthesia. Thirty minutes before surgery, rats were injected with the analgesic Ketofen (5 mg/kg; Merial, Velserbroek, The Netherlands) and the antibiotic Baytril (8.33 mg/kg; Bayer, Mijdrecht, The Netherlands). The local anesthetic 2% Xylocaine with adrenaline (10 mg/kg; Astra Zeneca, Zoetermeer, The Netherlands) was injected in the scalp, after which the skull was exposed and four burr holes were drilled and fitted with jeweler’s screws. Catheter tubing was tunneled from the scalp to an incision above the clavicle, where the catheter was inserted in the jugular vein and fixed in place using sterile sutures. A combination of 0.05 ml taurolidine-citrate solution (TCS; Access Technologies, Skokie, IL, USA) and a polyethylene cap was used to maintain catheter patency during the 1 week minimum period of recovery.

### Drugs

Cocaine (cocaine hydrochloride, OPG, Utrecht, The Netherlands) or nicotine (nicotine hydrogen tartrate salt, Sigma, St. Louis, MO, USA) was dissolved in sterile saline, and the pH of the solution was adjusted to 7.4 using sodium hydroxide solution. Both cocaine and nicotine solutions were sterilized through a 0.22 μm filter before self-administration.

### Summary of Experimental Timeline

Following at least 1 ‘week of recovery from surgery, rats were trained to self-administer either cocaine or nicotine over a period of 16 sessions (60 min/weekday). Extinction or retrieval-extinction sessions were conducted over a period of 16 days (70 min/weekday). Animals were then placed in their home cage for 34 days of abstinence, followed by a spontaneous recovery test session (60 min).


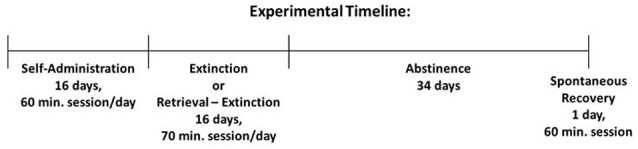


### Drug Self-administration

Rats were allowed to self-administer either cocaine or nicotine inside plexiglass operant chambers housed within sound-attenuating cubicles. Programs were executed using MED-PC hardware and software (Med Associates Inc., St. Albans, VT, USA). These operant chambers contained a fan for ventilation, two nose-poke holes, a red house light, a tone generator, a liquid swivel arm with polyethylene tubing protected by a metal spring and an infusion pump. Responding in the active nose-poke hole resulted in a drug infusion and presentation of a 15-s cue consisting of yellow light inside the nose-poke hole and a 3.5 kHz tone. For 15 s following a nose-poke in the active hole, responding in either the active or inactive nose-poke hole was without consequence (i.e., timeout period). Cocaine (500 μg/kg/infusion) or nicotine (40 μg/kg/infusion) was infused at a rate of 21 μl/s over 2 s. Responding in the inactive nose-poke hole had no effect, but was recorded.

Animals were first trained to self-administer nicotine or cocaine on a FR1 schedule for 10 daily sessions, followed by three sessions on an FR2 and three sessions on an FR4, for a total of 16 nicotine or cocaine self-administration sessions (each 60 min). To reduce cocaine overdose, the maximum number of rewards was set to 60 during the first six cocaine self-administration sessions. Catheter patency was maintained through a daily regimen of flushing catheters with a 0.05 ml solution of heparin (0.25 mg/ml) and gentamicin sulfate salt (0.08 mg/ml). At the end of the self-administration phase, catheter patency was tested by infusing 0.05 ml of the intravenous anesthetic thiopental sodium (50 mg/ml). Two rats were excluded from the cocaine experiment due to failed catheter patency, and one animal was excluded due to cocaine overdose.

### Retrieval and Extinction

Twenty-four hours following the final self-administration session, cocaine or nicotine rats were assigned to extinction vs. retrieval-extinction groups (total active and total inactive responding was counterbalanced between groups) and responding was extinguished during 16 daily (weekday) sessions of 70 min. During these sessions, the house light was turned on; cocaine or nicotine was unavailable, but response-contingent drug-conditioned cues were presented on an FR4 schedule of reinforcement. On each training day, the retrieval-extinction group was first placed in the operant chamber for a retrieval session of 10 min (Ret), then returned to their respective home cages for 10 min (Home). Afterward, rats were returned to the operant chamber for an additional 60 min of extinction training (Ext), which concluded the total daily training duration of 70 min. The extinction group was simply placed in the operant chamber for a contiguous 70 min training session without retrieval (No Ret). The first and second training days always occurred consecutively (i.e., no weekend gap). Following 16 training sessions, animals remained in their home cage for 34 days of abstinence.


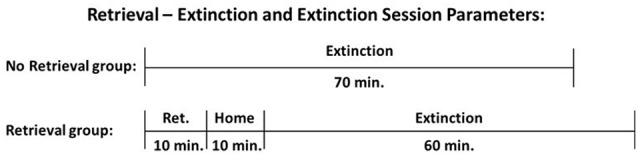


### Spontaneous Recovery

Following 34 days of home-cage abstinence, animals were returned to the operant chamber for a test of spontaneous recovery. This test was performed under extinction conditions (response-contingent drug cues presented on an FR4 schedule, drug unavailable) and lasted 60 min.

### Statistical Analyses

Behavioral data were analyzed using two-way repeated measures ANOVAs with experimental group as a between-subject factor and both session and nose poke as a within-subject factor. One cocaine rat was excluded due to high inactive nose pokes (>2 standard deviations from mean) during self-administration. Sidak’s *post hoc* comparisons were conducted when a significant interaction was observed. In some cases, one-way repeated measures ANOVAs were conducted to determine whether significant extinction occurred (for details, see “Results” section).

## Results

### Animals Successfully Acquired Nicotine or Cocaine Seeking

**Figure 1:**
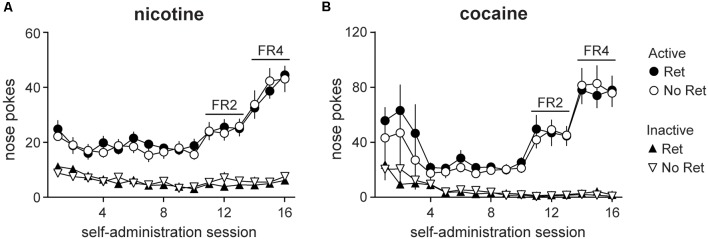
Self-administration of nicotine vs. cocaine. Nicotine **(A)** and cocaine **(B)** self-administration was conducted in daily sessions over weeks on an increasing FR schedule. Total active (circles) and inactive (triangles) lever presses on each daily session are depicted. Both retrieval (Ret) and no retrieval (No Ret) groups acquired drug seeking over days, and group assignments were appropriately matched on response rates (Nicotine Ret: *n* = 12; Nicotine No ret: *n* = 12; Cocaine Ret: *n* = 11; Cocaine No Ret: *n* = 9). All data are mean ± SEM.

Other than drug type, self-administration protocols for nicotine- vs. cocaine-seeking rats were identical. An increasing FR protocol was used to enhance rates of drug seeking and to verify successful acquisition of drug self-administration. Main effects of session were observed for both nicotine (Figure 1A) and cocaine (Figure 1B; nicotine: *F*_(4.71,103.62)_ = 30.33, *p* < 0.001; cocaine: *F*_(3.02,54.41)_ = 13.60, *p* < 0.001), as well as main effects of nose poke (active vs. inactive; nicotine: *F*_(1,22)_ = 201.16, *p* < 0.001; cocaine: *F*_(1,18)_ = 82.33, *p* < 0.001), and session * nose poke interactions (nicotine: *F*_(4.68,103.07)_ = 28.02, *p* < 0.001; cocaine: *F*_(3.45,62.19)_ = 16.81, *p* < 0.001). Whereas active nose pokes increased over sessions, inactive pokes decreased, indicating successful acquisition of drug seeking for both reinforcers. No group differences were detected, indicating that animals were appropriately balanced upon assignment to retrieval vs. no retrieval experimental groups.

### Both Nicotine and Cocaine Seeking Extinguish Over the Course of Repeated Retrieval

**Figure 2:**
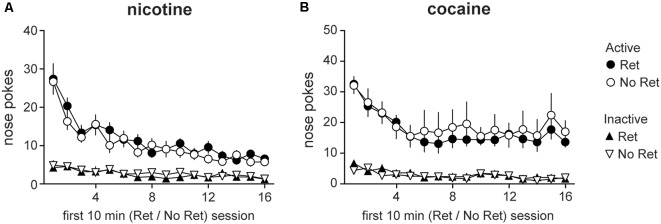
Drug seeking during the first 10 min of retrieval or no retrieval. Nicotine **(A)** and cocaine **(B)** seeking during retrieval vs. no retrieval. Total active (circles) and inactive (triangles) lever presses during the first 10 min of each daily retrieval-extinction session are depicted. Both retrieval (Ret) and no retrieval (No Ret) groups exhibited extinction over days, but there were no group differences (Nicotine Ret: *n* = 12; Nicotine No ret: *n* = 12; Cocaine Ret: *n* = 11; Cocaine No Ret: *n* = 9). All data are mean ± SEM.

After acquisition of drug self-administration, animals were assigned to experimental groups. The retrieval group underwent the first 10 min of extinction, then returned to the home cage for 10 min, and then completed the last 60 min of extinction. The no retrieval group underwent a contiguous 70 min extinction session. Both groups received response-contingent drug cues on an FR4 schedule, but no drug (for details, see “Materials and Methods” section). Main effects of session were observed for both nicotine (Figure 2A) and cocaine (Figure 2B) during the first 10 min of retrieval or no retrieval (nicotine: *F*_(5.92,130.32)_ = 29.26, *p* < 0.001; cocaine: *F*_(4.89,87.95)_ = 14.74, *p* < 0.001), indicating successful extinction of drug seeking. Main effects of nose poke (nicotine: *F*_(1,22)_ = 139.95, *p* < 0.001; cocaine: *F*_(1,18)_ = 47.44, *p* < 0.001) and session * nose poke interactions (nicotine: *F*_(5.23,115.14)_ = 20.16, *p* < 0.001; cocaine: *F*_(3.95,71.10)_ = 7.91, *p* < 0.001) were observed for both drugs as well. No group differences were detected.

### Retrieval Impairs Extinction of Nicotine, but Not Cocaine, Seeking

**Figure 3:**
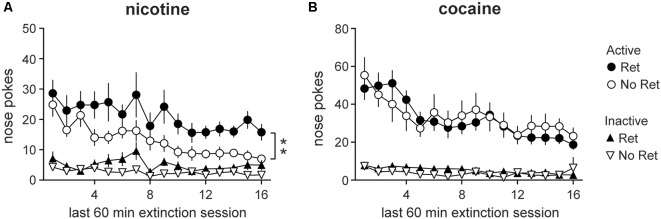
Drug seeking during the last 60 min of extinction. Nicotine **(A)** and cocaine **(B)** seeking during extinction. Total active (circles) and inactive (triangles) lever presses during the last 60 min of each daily extinction session are depicted. Both nicotine and cocaine-seeking animals exhibited extinction over days, but only nicotine-seeking animals showed a main effect of group. Nicotine-seeking in the retrieval (Ret) group was significantly higher than in the no retrieval (No Ret) group (Nicotine Ret: *n* = 12; Nicotine No ret: *n* = 12; Cocaine Ret: *n* = 11; Cocaine No Ret: *n* = 9). All data are mean ± SEM. **p < 0.01 main effect of group.

Analyses conducted over the last 60 min of extinction similarly revealed main effects of session for both nicotine (Figure 3A) and cocaine (Figure 3B; nicotine: *F*_(4.71,103.55)_ = 5.71, *p* < 0.001; cocaine: *F*_(6.21,111.85)_ = 9.44, *p* < 0.001), indicating successful extinction of drug seeking. Main effects of nose poke were found (nicotine: *F*_(1,22)_ = 96.36, *p* < 0.001; cocaine: *F*_(1,18)_ = 107.64, *p* < 0.001) as well as session * nose poke interactions (nicotine: *F*_(4.77,104.84)_ = 3.48, *p* = 0.007; cocaine: *F*_(6.00,107.97)_ = 6.64, *p* < 0.001) for both drugs. However, a main effect of group was also detected for nicotine seeking (*F*_(1,22)_ = 11.42, *p* = 0.003) as well as a nose poke * group interaction (*F*_(1,22)_ = 4.42, *p* = 0.047), indicating nicotine seeking was higher in the retrieval group than in the no retrieval group. To determine whether groups showed equivalent extinction over days, one-way repeated measures ANOVAs were conducted on active nose pokes. A main effect of session was detected in the no retrieval group (*F*_(1,15)_ = 4.67, *p* = 0.001), but not the retrieval group (*F*_(1,15)_ = 1.92, *p* = 0.150), suggesting that extinction was impaired in the retrieval group.

### Retrieval Promotes Spontaneous Recovery of Nicotine, but Not Cocaine, Seeking

**Figure 4:**
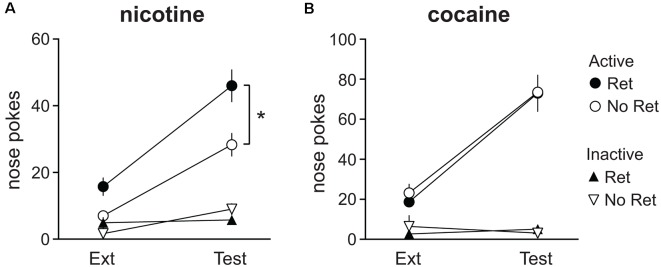
Spontaneous recovery of drug seeking after abstinence. Nicotine **(A)** and cocaine **(B)** seeking at the end of extinction (Ext) training vs. the spontaneous recovery test (Test). Total active (circles) and inactive (triangles) lever presses during the last 60 min of Ext is depicted, next to responding on the 60 min Test. Both cocaine- and nicotine-seeking animals exhibited spontaneous recovery. Nicotine-seeking in the retrieval (Ret) group was significantly higher than in the no retrieval (No Ret) group (Nicotine Ret: *n* = 12; Nicotine No ret: *n* = 12; Cocaine Ret: *n* = 11; Cocaine No Ret: *n* = 9). All data are mean ± SEM. *p < 0.05 main effect of group.

To assess the effects of the retrieval-extinction procedure on spontaneous recovery of drug seeking, the last 60 min of extinction training (on the last extinction session) was compared to responding after 34 days of abstinence during the spontaneous recovery test (also 60 min). Main effects of session were observed for both nicotine (Figure 4A) and cocaine (Figure 4B; nicotine: *F*_(1,22)_ = 36.39, *p* < 0.001; cocaine: *F*_(1,18)_ = 68.39, *p* < 0.001) as well as main effects of nose poke (nicotine: *F*_(1,22)_ = 106.11, *p* < 0.001; cocaine: *F*_(1,18)_ = 146.99, *p* < 0.001) and session * nose poke interactions (nicotine: *F*_(1,22)_ = 34.52, *p* < 0.001; cocaine: *F*_(1,18)_ = 71.84, *p* < 0.001), indicating spontaneous recovery of drug seeking. Additionally, a main effect of group (*F*_(1,22)_ = 6.66, *p* = 0.017) and nose poke * group interaction (*F*_(1,22)_ = 6.35, *p* = 0.020) was detected for nicotine seeking, indicating nicotine seeking was higher in the retrieval group than in the no retrieval group. No group differences were observed for cocaine. Thus, the retrieval-extinction procedure promoted nicotine seeking during both extinction and spontaneous recovery, but did not alter cocaine seeking.

## Discussion

This study revealed a heretofore unreported and paradoxical detrimental effect of retrieval-extinction memory reactivation procedures on extinction of nicotine seeking, concomitant with enhanced spontaneous recovery of nicotine seeking. To our knowledge, this is the first report of detrimental effects using a standard retrieval-extinction procedure on any conditioned behavior. Others have reported null effects of the retrieval-extinction procedure (Chan et al., 2010; Costanzi et al., 2011), similar to our findings on cocaine seeking reported here. Ma et al. (2012) reported detrimental effects of retrieval-extinction on morphine conditioned place preference (i.e., enhanced spontaneous recovery), but only with a very long interval between retrieval and extinction (3 h) which is outside the expected reconsolidation window, and could have interfered with extinction memory consolidation (Tronson and Taylor, 2007; Monfils et al., 2009). Millan et al. (2013) reported enhanced reacquisition of alcohol seeking and increased motivation to seek alcohol under progressive ratio schedules after an inverted extinction-retrieval procedure (i.e., extinction first, then retrieval), but this same procedure also reduced context-induced reinstatement of alcohol seeking (Millan et al., 2013). The latter study underscores the possibility that similar behavioral manipulations can be both therapeutic and detrimental, depending on the outcome measure. Here, we chose to focus on spontaneous recovery of drug seeking because this allowed us to specifically examine the effects of retrieval-extinction on long-term drug seeking triggered by the conditioned drug cues (tone + light). In addition, spontaneous recovery is a commonly used outcome measure for the efficacy of retrieval-extinction procedures (Monfils et al., 2009; Flavell et al., 2011; Ma et al., 2012; Xue et al., 2012; Kredlow et al., 2016).

In this study, we compared behavioral effects of retrieval-extinction procedures using two different reinforcers, nicotine and cocaine. Despite their different pharmacological mechanisms of action, both drugs result in an accumulation of dopamine in the nucleus accumbens during drug exposure, which is thought to underlie their rewarding and reinforcing effects (Balfour et al., 2000; Fadda et al., 2003; Dong et al., 2013). Dopamine is also important for encoding drug-conditioned cue associations, and over time with repeated drug use, these cues acquire incentive salience and the ability to promote, or sustain, drug seeking even under extinction conditions (i.e., when drug is no longer available; Bevins and Palmatier, 2004).

Drug-conditioned cues are essential for memory reactivation during retrieval, and the ability of memory reactivation to trigger reconsolidation is thought to be a critical determinant of retrieval-extinction therapeutic efficacy (Auber et al., 2013; Olshavsky et al., 2013). That is, the reconsolidation process must be initiated during retrieval in order for memory-updating to occur during the subsequent extinction phase (Monfils et al., 2009). The duration of retrieval session (10 min) in the present study was comparable to other studies that have demonstrated therapeutic success with retrieval-extinction procedures (Flavell et al., 2011; Xue et al., 2012). Nonetheless, it is possible that we failed to trigger reconsolidation with our protocol. Given that the ability to induce reconsolidation is inversely related to the strength of the memory trace (Eisenberg et al., 2003; Tronson and Taylor, 2007), the cue memories for cocaine and nicotine might have been particularly strong in our paradigm. Because retrieval-extinction produced an effect, albeit a detrimental one, in nicotine rats, another explanation is that reconsolidation was effectively triggered, and extinction training paradoxically strengthened the retrieved cue memory.

In line with the latter interpretation, retrieval impaired extinction of nicotine seeking. This extinction impairment must be attributed to the retrieval-extinction procedure since extinction success was observed in the contiguous extinction group. Thus, retrieval-extinction induces extinction failure in nicotine rats under these conditions. Why might this occur? There are some notable distinctions in nicotine-conditioned cues that are worth considering. Whereas nicotine alone is a relatively weak reinforcer, it is very effective at enhancing the incentive salience, and reinforcing properties, of cues paired with nicotine (Chaudhri et al., 2006; Caggiula et al., 2009). In rats that acquire nicotine self-administration paired with conditioned cues similar to the ones used in the present study, cessation of the nicotine conditioned-cue availability leads to faster extinction than cessation of nicotine availability (Caggiula et al., 2001). Furthermore, acquisition of nicotine self-administration is impaired if such nicotine conditioned cues are never available (Caggiula et al., 2002; Cohen et al., 2005).

The aforementioned findings have led some to propose that nicotine-seeking behavior is primarily maintained not by nicotine itself but by its ability to enhance the reinforcing properties of both pharmacological and non-pharmacological (i.e., cues) stimuli (Chaudhri et al., 2006; Caggiula et al., 2009). This hypothesis is supported by observations that rats will respond more for cues in the presence of nicotine, even when the cues and nicotine are self-administered on separate levers (Palmatier et al., 2006). Cocaine, by contrast, is a much more powerful primary reinforcer (Risner and Goldberg, 1983), and rats will readily acquire cocaine self-administration in the absence of cocaine-associated cues (Fuchs et al., 2006). The relative importance of the drug-conditioned cues vs. the primary reinforcer (nicotine vs. cocaine) may thus be a key factor in the differences we observed with the retrieval-extinction procedure (Bevins and Palmatier, 2004). The greater ability of nicotine-conditioned cues to sustain nicotine seeking may have resulted in a paradoxical updating of the memory with “sustained seeking” as opposed to “extinction” memory trace.

We observed enhanced spontaneous recovery and impaired extinction after retrieval-extinction procedures in nicotine, but not cocaine rats. It is important to note, however, that the increase in spontaneous recovery may be attributed, at least in part, to the extinction failure induced by retrieval-extinction in nicotine rats. Because we were unable to fully extinguish nicotine seeking after retrieval-extinction, the two experimental groups (Ret vs. No Ret) were tested for spontaneous recovery despite their pre-existing difference in extinction baseline. Additional experiments are necessary to determine whether the Ret group would eventually extinguish nicotine seeking to levels achieved in the No Ret group and if spontaneous recovery would remain elevated in the Ret group under such circumstances. Furthermore, additional studies should extend these findings to other measures of nicotine seeking, such as renewal, reinstatement (e.g., after a nicotine priming injection), and reacquisition of nicotine seeking.

The lack of effect of retrieval-extinction procedures on cocaine seeking differs from that reported by Xue et al. (2012). Retrieval-extinction enhanced the rate of extinction of cocaine seeking, although the magnitude of this effect differed across three independent experiments. In each experiment, however, retrieval-extinction procedures attenuated the reinstatement, renewal, or spontaneous recovery of cocaine seeking. This was in contrast to the complete blockade of cocaine conditioned place preference, which relies on purely Pavlovian drug-cue associations. Indeed, there is some evidence that operant, response-outcome memories may be less prone to undergo reconsolidation (Hernandez and Kelley, 2004). However, even well-trained instrumental behaviors have been shown to undergo reconsolidation (Exton-McGuinness et al., 2014), including nicotine- and cocaine-seeking (Tedesco et al., 2014; Sorg et al., 2015). It is also worth noting that the unconditioned stimulus, nicotine or cocaine itself, can be an effective trigger for reconsolidation (Xue et al., 2017; Zhu et al., 2018), although we did not employ this strategy in the present study. Other potential differences that could account for the apparent discrepancy between this study and Xue et al. (2012) include the cocaine-taking history of the animals, cocaine dose, duration of abstinence, training schedule of reinforcement, and duration of cue presentations and extinction sessions. Clearly, more work is needed to fully understand the boundary conditions limiting the efficacy of retrieval-extinction procedures.

Though the retrieval-extinction procedures used in the present study induced unexpected increases in nicotine seeking, some positive outcomes have been reported using other retrieval-extinction procedures in rats (Auber et al., 2014) and human smokers (Germeroth et al., 2017). In contrast with the present study, which used response-contingent nicotine cues during retrieval in the same context where nicotine was taken, both of the aforementioned studies used non-contingent (i.e., passive) nicotine-cue exposures during the retrieval phase, and retrieval was conducted in a context that was different from the one where nicotine was taken (Auber et al., 2014; Germeroth et al., 2017). Interestingly, Xue et al. (2017) used nicotine (as opposed to nicotine cues) to trigger retrieval and was able to demonstrate reductions in nicotine seeking in rats, as well as nicotine craving in human smokers after disrupting reconsolidation with propranolol. Germeroth et al. (2017) observed both reductions in craving in response to nicotine cues after retrieval-extinction, as well as a reduction in the average number of cigarettes smoked per day over a 2-week and 1-month follow up. However, retrieval-extinction did not alter physiological responses to nicotine cues, relapse, or days abstinent (Germeroth et al., 2017). Thus, certain components of the nicotine memory may be more receptive to retrieval-extinction therapy than others, and the method in which retrieval is triggered may be an important variable. While it may be possible to weaken some aspects of the nicotine memory, our data suggest it is also possible to strengthen nicotine seeking with retrieval-extinction under certain conditions.

These findings add to a growing literature indicating highly variable effects of retrieval-extinction procedures across both preclinical and clinical models (Auber et al., 2013; Kindt and Soeter, 2013; Millan et al., 2013). Future work should continue with a careful examination of the boundary conditions determining the outcome of these memory reactivation procedures, which were originally identified as therapeutic (Monfils et al., 2009; Schiller et al., 2010). We hypothesize that the detrimental effects of retrieval-extinction procedures on nicotine seeking may be due to the fact that nicotine-associated cues are so essential to the reinforcing properties of nicotine, perhaps even more than nicotine itself (Caggiula et al., 2001; Bevins and Palmatier, 2004; Palmatier et al., 2006). Given the high rates of smoking and vaping nicotine amongst people who abuse psychostimulants and other substances (Tzilos et al., 2016; Temple et al., 2017), the implementation of retrieval-extinction procedures in humans should be undertaken with great care.

## Data Availability Statement

All datasets generated for this study are included in the manuscript.

## Ethics Statement

The animal study was reviewed and approved by the Animal Experiments Committee of the VU University, Amsterdam, The Netherlands.

## Author Contributions

RS, JP, and TV designed the experiments, interpreted the data, contributed intellectually to the project, edited and finalized the manuscript. RS and JP drafted the manuscript. RS acquired and analyzed the data.

## Conflict of Interest

The authors declare that the research was conducted in the absence of any commercial or financial relationships that could be construed as a potential conflict of interest.
